# Biochemical Analysis of Leukocytes after In Vitro and In Vivo Activation with Bacterial and Fungal Pathogens Using Raman Spectroscopy

**DOI:** 10.3390/ijms221910481

**Published:** 2021-09-28

**Authors:** Aikaterini Pistiki, Anuradha Ramoji, Oleg Ryabchykov, Daniel Thomas-Rüddel, Adrian T. Press, Oliwia Makarewicz, Evangelos J. Giamarellos-Bourboulis, Michael Bauer, Thomas Bocklitz, Jürgen Popp, Ute Neugebauer

**Affiliations:** 1 Leibniz Institute of Photonic Technology Jena (a Member of Leibniz Health Technologies), Albert-Einstein-Straße 9, 07745 Jena, Germany; aikaterini.pistiki@uni-jena.de (A.P.); anuradha.ramoji@med.uni-jena.de (A.R.); Oleg.ryabchykov@uni-jena.de (O.R.); thomas.bocklitz@uni-jena.de (T.B.); juergen.popp@leibniz-ipht.de (J.P.); 2 Center for Sepsis Control and Care, Jena University Hospital, Am Klinikum 1, 07747 Jena, Germany; daniel.thomas@med.uni-jena.de (D.T.-R.); adrian.press@med.uni-jena.de (A.T.P.); michael.bauer@med.uni-jena.de (M.B.); 3 Institute of Physical Chemistry and Abbe Center of Photonics, Friedrich Schiller University, Helmholtzweg 4, 07743 Jena, Germany; 4 Department of Anesthesiology and Intensive Care, Jena University Hospital, Am Klinikum 1, 07747 Jena, Germany; 5 Medical Faculty, Friedrich Schiller University, Bachstraße. 18, 07743 Jena, Germany; 6 Institute of Infectious Diseases and Infection Control, Jena University Hospital, Am Klinikum 1, 07747 Jena, Germany; oliwia.makarewicz@med.uni-jena.de; 7 4th Department of Internal Medicine, Medical School, National and Kapodistrian University of Athens, Rimini Street 1, 12462 Athens, Greece; egiamarel@med.uoa.gr; 8 Jena Biophotonics and Imaging Laboratory, Albert-Einstein-Straße 9, 07745 Jena, Germany

**Keywords:** Raman microspectroscopy, leukocytes, infection model, *Staphylococcus aureus*, *Klebsiella pneumoniae*, *Candida albicans*, neutrophil, PBMC, monocyte, lymphocyte

## Abstract

Biochemical information from activated leukocytes provide valuable diagnostic information. In this study, Raman spectroscopy was applied as a label-free analytical technique to characterize the activation pattern of leukocyte subpopulations in an in vitro infection model. Neutrophils, monocytes, and lymphocytes were isolated from healthy volunteers and stimulated with heat-inactivated clinical isolates of *Candida albicans*, *Staphylococcus aureus*, and *Klebsiella pneumoniae*. Binary classification models could identify the presence of infection for monocytes and lymphocytes, classify the type of infection as bacterial or fungal for neutrophils, monocytes, and lymphocytes and distinguish the cause of infection as Gram-negative or Gram-positive bacteria in the monocyte subpopulation. Changes in single-cell Raman spectra, upon leukocyte stimulation, can be explained with biochemical changes due to the leukocyte’s specific reaction to each type of pathogen. Raman spectra of leukocytes from the in vitro infection model were compared with spectra from leukocytes of patients with infection (DRKS-ID: DRKS00006265) with the same pathogen groups, and a good agreement was revealed. Our study elucidates the potential of Raman spectroscopy-based single-cell analysis for the differentiation of circulating leukocyte subtypes and identification of the infection by probing the molecular phenotype of those cells.

## 1. Introduction

Leukocytes are part of the human immune system and play a very important role in recognizing and fighting infections. The various immune cells have different tasks, including elimination of the pathogen by phagocytosis or release of mediators. During infection, interaction of the pathogen with the host leads to activation of immune cells and often to a change in the relative ratio of leukocyte subpopulations. Therefore, differential white blood cell count is one of the routine methods performed for infection diagnostics [1]. In addition, various protein-based biomolecules such as, e.g., procalcitonin, C-reactive protein, and interleukin-6, are utilized within clinics for diagnostics and to determine the patient’s inflammatory state [2,3]. For most biomarkers, a separate assay has to be performed [4]. Despite developing new clinical and laboratory tools, the timely diagnosis of infection and the causative pathogen remains challenging. A further in-depth understanding of biochemical changes in the immune cells in response to infection could help to pave the way for new diagnostic methods [5,6]. Especially the identification of the infection-causing pathogen remains time consuming when cultivation-based microbiological methods are used. Detailed information on the infectious agent is usually only available within 24 to 48 h, but might take even longer in case of uncommon or fastidious pathogens [7,8]. The extended time-to-results forces the treating physician to use empirical antibiotic treatment, which is not always appropriate for eliminating the pathogen and leads to high resistance rates, especially in nosocomial infections. [9] Thus, a diagnostic test that can provide valuable information not only about the patient’s condition, but also about the type (bacterial or fungal) and status of infection within the first hours is urgently needed. Tremendous effort is being made to develop new diagnostic methods to speed up diagnosis time. New methods include mass spectrometry and molecular analysis, but also vibrational spectroscopy [8,10,11]. The latter is also the method utilized in this work.

In the last decade, label-free photonic methods provided promising results in characterizing leukocytes after minimal sample preparation [11]. In particular, Raman spectroscopy—a label-free and non-destructive vibrational spectroscopic method—can provide valuable insights into the biochemical profiles of single biological cells [12,13]. The Raman spectra capture vibrational mode information of the biomolecules present in the cell. Analysis of these vibrational modes provides information on the cell’s chemical composition that can be translated afterward into biochemical profiles of the measured cells. Leukocyte subpopulations can be identified and differentiated via Raman spectroscopy based on their spectroscopic fingerprints in clinical samples. [14,15,16,17,18] Further Raman spectroscopy detected immune cell activation and apoptosis. [19,20,21,22,23,24,25] Recently, in vitro stimulation studies with neutrophils pointed to the potential of Raman spectroscopy to differentiate the cause of infection [26]. Also, a first study highlighted the translational potential of Raman spectroscopic leukocyte characterization for patient stratification [27].

The aim of this study was to capture and describe the distinct immune response of leukocyte sub-populations to different pathogens using Raman spectroscopy. For this purpose, an in vitro infection model was designed using leukocytes from healthy donors. Further, the spectral features captured in the in vitro infection model were compared to those observed in in vivo activated leukocytes isolated from patients with infection in order to investigate the translational potential of Raman spectroscopy for infection analysis.

## 2. Results

### 2.1. Design and Biological Characterization of the In Vitro Infection Model

**Figure 1:**
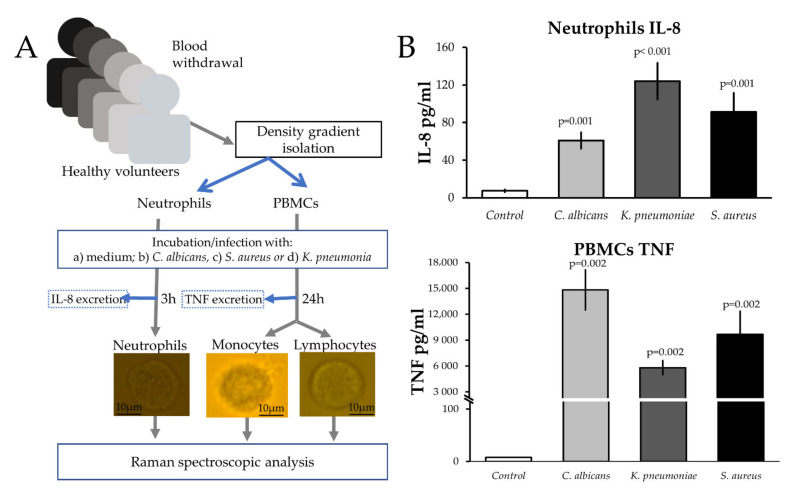
(**A**). Schematic depicts an experimental flow. (**B**). Concentrations of IL-8 in neutrophils and TNF in PBMCs supernatants (mean ± SE). *p*-values show a statistical difference compared with the control group. There is no error bar for the control treatment of TNF because all measured concentrations were below the limit of detection.

Figure 1A schematically depicts the experimental workflow of the cell stimulation in this study. Cytokine measurements in the supernatants confirm cell-stimulation by the heat-killed pathogens, showing higher cytokine concentrations in stimulated cells than in unstimulated cells (Figure 1B). Heat-killed pathogens were used to contribute only from immune cells and towards the biochemical changes occurring during cellular activation processes. The pattern shown in Figure 1B of TNF and IL8 released by peripheral blood mononuclear cells (PBMCs) and neutrophils, respectively, after stimulation with fungi, Gram-positive and Gram-negative bacteria, agrees with previously published results [28,29,30,31].

### 2.2. Influence of Donor Variability on Raman Spectral Features

As an initial step, the influence of donor variability on Raman spectra of the leukocytes was examined. Mean spectra for the three sub-populations are shown in Supplementary Figure S1. Principal component analysis (PCA), shown in Supplementary Figure S2A, revealed discrimination of untreated neutrophils, lymphocytes, and monocytes besides the donor-to-donor variations analysis. The PCA loadings highlight the Raman bands, wherein leukocyte subtype-specific spectral differences are to be found. Despite high donor-to-donor variability it can be observed that the variability between cell subpopulations is consistent for the entire dataset. The results shown in Supplementary Figure S2B are in agreement with our previous results. [14,15]

### 2.3. Differentiation between Infected and Non-Infected Leukocyte Subpopulations in In Vitro Infection Models

**Figure 2:**
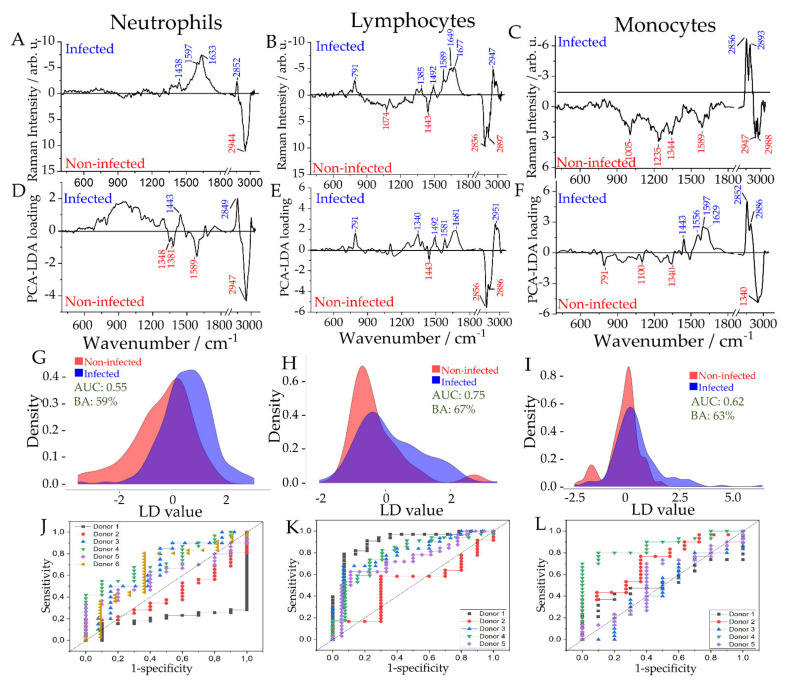
Raman spectroscopic analysis of in vitro stimulation of leukocyte subpopulations and statistical analysis to differentiate infected and non-infected cells. Each leukocyte subpopulation was infected individually with the heat-inactivated pathogens: *K. pneumoniae*, *S. aureus*, and *C. albicans*. Analysis of the infected cells was performed collectively for each leukocyte subpopulation. Raman difference spectra (**A**–**C**), PCA loadings (**D**–**F**), distribution of fitted LD model scores (**G**–**I**), and ROC curves for the cross-validated predictions (**J**–**L**) are given for neutrophils (**A**,**D**,**G**,**J**), lymphocytes (**B**,**E**,**H**,**K**) and monocytes (**C**,**F**,**I**,**L**). The ROC curves (**J**–**L**) are calculated for each donor. Achieved balanced accuracy (BA) on the sample level and average AUC on the cell level are used as metrics for the PCA-LDA models are given as insets to the distribution of fitted LD model scores (**G**–**I**).

Spectra were categorized into a “non-infected” group that included the Raman spectra of non-stimulated cells, and an “infected” group. The “infected” group included Raman spectra of all cells of the respective subpopulations, exposed to one of three different pathogens *S. aureus*, *K. pneumoniae*, or *C. albicans*. Supplementary Figure S1 depicts the average Raman spectra of leukocyte subpopulations. Raman difference spectra of the non-infected and infected group were computed for each subpopulation to reveal activation-induced biochemical changes. Classification models to differentiate the non-infected group vs. the infected group were built separately for neutrophils, lymphocytes, and monocytes. Loadings, score plots and receiver operating characteristic (ROC) curves, and area under the ROC curves (AUC) are depicted in Figure 2. As depicted by the LD vector (Figure 2D–F), some of the Raman bands used by the PCA-LDA model are observed in the Raman difference spectra indicating similarity in the biochemical composition captured by the PCA-LDA loadings and the difference spectra (Figure 2A–C). The difference spectra are calculated by subtracting the average spectrum of one group from the other. Thus, background information present in both groups (e.g., water) should cancel out. The PCA-LDA model is optimized in the cross-validation routine to classify previously unseen data. The PCA-LDA loadings depict the differences which are important for the classification and are less influenced by the donor-to-donor variations. However, due to the small sample size it is important to evaluate both plots to verify the spectral differences. Raman band assignment is given in Supplementary Table S1.

Most of the studies related to neutrophils address the early phase (~1 h of stimulation) of infection. In this study, we were interested in looking at the changes occurring at the later phase (~3 h) of activated neutrophils. Known changes include transcriptional activation as well as resolution phase or chemokine receptor expression [32,33]. The balanced accuracy of the Raman model reaches only 59% (Figure 2D), which might be caused by the heterogeneous immune cell activation through bacteria or fungi. The neutrophil’s responses to pathogens are complex and known to be microbe-dependent [33], also reflected in different Raman signals [26]. In addition, donor-to-donor variations might have an influence. Besides a different response mechanism due to pathogen size, also different receptors are activated [34]. For large fungal cells and some aggregating bacteria, neutrophils form neutrophil extracellular traps (NETs), which have not been reported in response to *K. pneumoniae* infection [34]. This heterogeneity in neutrophil’s response to the different pathogens might be a reason why the detection of a typical activation pattern in the infection group is difficult. We also observe high heterogeneity in neutrophils response to bacterial infections (see PCA scatterplots in Figures S3A, S4A and S5A). Furthermore, a donor-to-donor variation is observed, as can be seen in Figure 2J. Nevertheless, the difference spectrum generated by subtracting Raman spectra of non-infected cells from infected cells reveals spectral differences; Raman bands around 2950 cm^−1^ might indicate overall changes in the protein expression (Supplementary Table S1, Figure 2A). Similar differences are also visible in the LD loading of the classification model (Figure 2D).

ROC curves show satisfactory differentiation between infected and non-infected lymphocytes with an average AUC of 0.75 and balanced accuracy of 67% (Figure 2K). This is consistent with the PCA score plot in Figure S3C. Lymphocytes, after in vitro infection, show a relative increase in the intensity of the Raman bands at 791, 1340/1344, 1492, and 1581/1589 cm^−1^, indicating a relative increase in the nucleic acid content of the cells [23,35,36]. Higher band intensity of CH-stretching vibration at 2947/2951 cm^−1^ and amide I at 1649 cm^−1^ indicate higher protein content in the infected cells than non-infected cells. Increased nucleic acid and protein content can be associated with increased transcriptomic activity upon pathogen-induced activation [37]. The sharp Raman bands at 1443, 2856, 2886, and 2897 cm^−1^ might point to the higher lipid content in non-infected lymphocytes. The reduced lipid content in activated cells could be explained by exocytosis and cytokine release [38].

Activated and nonactivated monocytes can be distinguished with an average AUC of 0.65 with a balanced accuracy of 63% (Figure 2L). Only a weak differentiation can be seen in the PCA scatter plot in Figure S3E. In monocytes, changes in nucleic acid composition in infected and non-infected cells can be assigned based on the phosphate backbone vibration at 791 cm^−1,^ and other nucleic acid vibrations at 1100, 1340/1344, 1589, and 1597 cm^−1^ are observed, indicating a difference in the nucleic acid composition from both DNA and RNA. The intensity of all the bands assignable to proteins appears with lower intensity in the infected cells (1005, 1235, 1556, 1629, and 2947/2951 cm^−1^). Higher Raman bands at 2852/2856 and 1443 cm^−1^ (CH_2_CH_3_ deformation) might point to an increase of lipid content in infected monocytes. The observed changes in nucleic acids, protein, and lipid-associated vibrations could be explained by these cells’ cytokine release and phagocytic activity when exposed to the pathogens [39,40].

### 2.4. Differentiation of Leukocyte Sub-Populations Activated by Bacteria and Fungi

**Figure 3:**
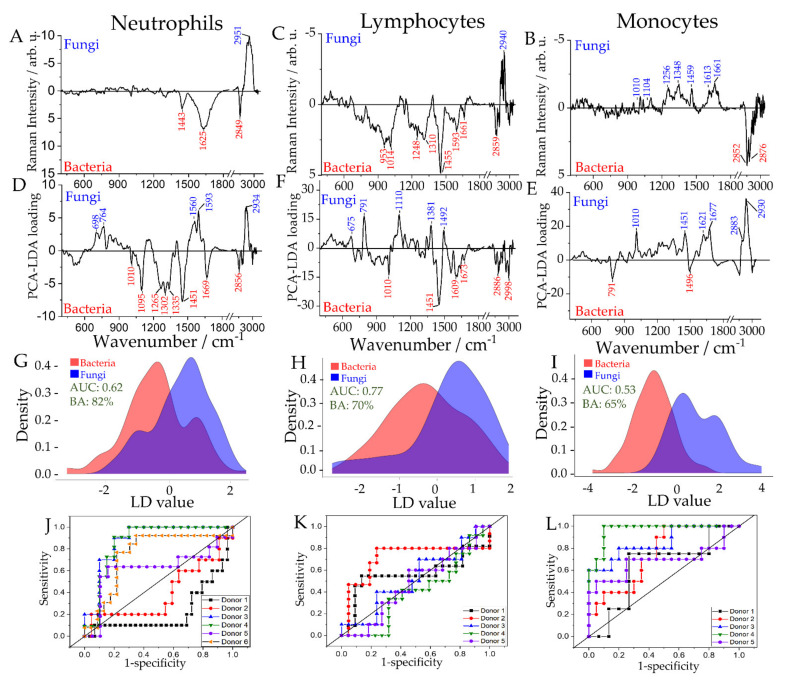
Raman spectroscopic analysis of in vitro stimulation of leukocyte subpopulations to differentiate fungal and bacterial infection. Raman spectra of pure subpopulations were recorded after neutrophils’ and PBMCs’ infection with *K. pneumoniae*, *S. aureus*, or *C. albicans* (Figure S1). Raman difference spectra (**A**–**C**), PCA loadings (**D**–**F**), distribution of fitted LD model scores (**G**–**I**), and ROC curves for the cross-validated predictions (**J**–**L**) are given for neutrophils (**A**,**D**,**G**,**J**), lymphocytes (**B**,**E**,**H**,**K**) and monocytes (**C**,**F**,**I**,**L**). The ROC curves (**J**–**L**) are calculated individually for each donor. Achieved balanced accuracy (BA) on the sample level and average AUC on the cell level for the PCA-LDA models are given as insets to the distribution of fitted LD model scores (**G**–**I**).

Next, we asked the question of whether it is possible to discriminate between cells challenged with fungi (*C. albicans*) and cells incubated with bacterial pathogens (*K. pneumoniae* and *S. aureus*). Figure 3 shows the computed Raman difference spectra and statistical models for all three investigated leukocyte sub-populations. The first two PCs for each leukocyte subpopulation are shown in Figure S4A,C,E. ROC curves differentiated between the two groups for all leukocyte sub-types: an average AUC of 0.62, at the cell level, with a balanced accuracy of 82%, at the sample level, for neutrophils, an average AUC of 0.53, and balanced accuracy of 65% for the lymphocytes and an average AUC of 0.77 with a balanced accuracy of 70% percent for monocytes (Figure 3D–F, respectively). A detailed Raman band assignment is found in Supplementary Table S2.

A more pronounced amide I band (1669 cm^−1^) and differences in the symmetric CH-stretching (2934 cm^−1^) and asymmetric CH-stretching (2951 cm^−1^) bands indicate differences in the protein composition of neutrophils after stimulation with fungi and bacteria. Also, Raman bands associated with nucleic acids show up in the PCA-LDA loading plot (e.g., 1095 and 1593 cm^−1^) and differences in the C-H deformation band at 1451 cm^−1^. Different response of neutrophils to bacteria and much larger fungi has been reported previously [34,41].

The AUC for lymphocytes is low ~0.53; however, the LD vector indicates significant differences in the biochemical composition of lymphocytes activated by fungi/bacteria. Differences in nucleic acid composition are observed (791 and 1496 cm^−1^ when infected with bacteria and 1104 and 1348 cm^−1^ when infected with *Candida*). Furthermore, spectral differences are observed in vibrational bands associated with proteins (1010, 1256, 1613, 1621, 1661, and 2930 cm^−1^), as well as in vibrations assigned to C–H deformation (1451/1459 cm^−1^) and C–H stretching (2852 and 2876 cm^−1^) indicating variations in the relative of the content of proteins, carbohydrates, and lipids upon stimulation with bacteria or fungi, respectively.

In the PCA–LDA loading plot and the computed Raman difference spectrum of differently stimulated monocytes, pronounced bands indicate a different response of this cell population to the two stimuli. The Raman bands are assigned to nucleic acids (675, 791, 953, 1100, 1381, 1593 cm^−1^), proteins (1010/1014, 1248, 1609, 1661 cm^−1^), lipid (2859, 2886, 2998 cm^−1^) as well as a mixture of proteins, lipids, and carbohydrates (1310, 1451/1455 cm^−1^). The pathogen-specific activation of monocyte’s defense functions, including the production of reactive oxygen and nitrogen species, as well as direct uptake and killing, has been reported previously [42].

These results reveal that neutrophils and monocytes, although having a common goal of disabling pathogens, respond differently depending on the invading pathogen [43], and these differences in response can be detected by Raman spectroscopy.

### 2.5. Differentiation of Leukocyte Sub-Populations Activated by Gram-Positive and Gram-Negative Bacteria

**Figure 4:**
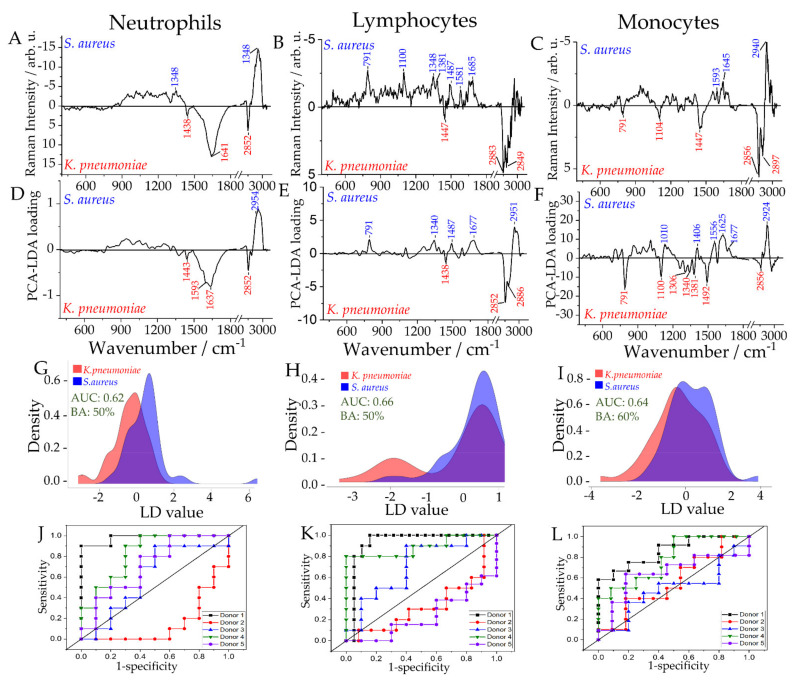
Raman spectroscopic analysis of in vitro stimulation of leukocyte subpopulations to differentiate Gram-positive and Gram-negative bacterial infections. Raman spectra of pure subpopulations were recorded from neutrophils and PBMCs infected with Gram-negative *K. pneumoniae* or Gram-positive *S. aureus*. Raman difference spectra (**A**–**C**), PCA loadings (**D**–**F**), distribution of fitted LD model scores (**G**–**I**) and ROC curves for the cross-validated predictions (**J**–**L**) are given for neutrophils (**A**,**D**,**G**,**J**), lymphocytes (**B**,**E**,**H**,**K**) and monocytes (**C**,**F**,**I**,**L**). In addition, the balanced accuracy (BA) on the sample level and average AUC on cell level for the distribution of fitted LD model scores (**G–I**).

As a step further, Raman models were built to differentiate between cells infected with Gram-positive and Gram-negative bacteria. The balanced accuracy obtained from Raman models for all the investigated cell populations to differentiate between the Gram-positive and Gram-negative infected cells was low. Low differentiation between the groups was also observed in PCA scatterplots in Figure S5A,C,E. Neutrophils and lymphocytes provided a balanced accuracy below 60% and were therefore not further discussed. However, difference spectra and LD loading vectors of neutrophils and lymphocytes infected with Gram-positive and Gram-negative bacteria are shown in Figure 4, for the sake of completeness.

The Raman model for the monocyte population provided an AUC of 0.64 and balanced accuracy of 60%. Despite the relatively low balanced accuracy, the Raman difference spectra and the LDA loading show prominent differences present between the Raman spectra of monocytes activated by representatives of Gram-positive bacteria and Gram-negative bacteria. It can be seen that upon infection with the Gram-negative pathogen (*K. pneumoniae*), an increase in the bands at 791, 1104/1100, 1340, and 1492 cm^−1^ is observed, indicating a relative increase of nucleic acids (Table S3). The increase of 2856 and 2897 cm^−1^ Raman bands, the CH_2_CH_3_ deformation band (1447 cm^−1^), and the CH-stretching at 1306 cm^−1^ indicate chemical differences in the lipid, protein, and carbohydrate composition. In cells infected with the Gram-positive pathogen *S. aureus,* a relative increase in the Raman bands at 1010, 1556, 1625, 1645, 2924, and 2940 cm^−1^ is observed, indicating a relative increase in protein content. These spectral differences reflect the different responses of monocytes towards Gram-positive and Gram-negative pathogens, especially in lipid and protein profiles [44,45,46,47].

### 2.6. Comparison of Spectral Features Observed in In Vitro Models with Patient Samples

**Figure 5:**
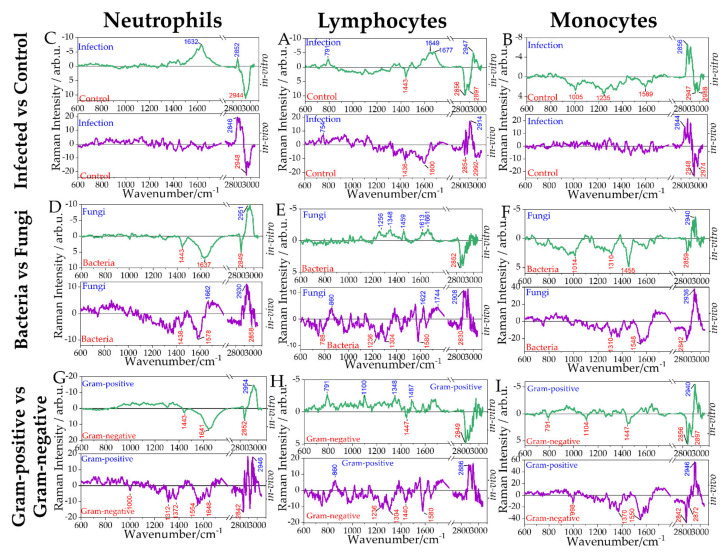
Comparison of Raman difference spectra of in-vivo-activated leukocytes against in-vitro-activated leukocytes. The in-vivo-activated leukocytes were isolated from patients having sterile inflammation (*inflammation due to non-infectious origin*) and infection/sepsis. The in-vitro-activated leukocytes subtypes were isolated from healthy donors. Raman difference spectra of neutrophils (**A**) infected/control, (**D**) bacterial/fungi and (**G**) Gram-positive bacteria/Gram-negative bacteria. Raman difference spectra of lymphocytes (**B**) infected/control, (**E**) bacterial/fungi, and (**H**) Gram-positive bacteria/Gram-negative bacteria. Raman difference spectra of monocytes (**C**) infected/control, (**F**) bacterial/fungi, and (**I**) Gram-positive bacteria/Gram-negative bacteria. Assignment of the Raman bands is given in Table S1. It has to be noted that there were no patients with pure fungal infection (Supplemental Table S4). Thus, panels (**D**–**F**) have to be read with care.

Raman spectroscopic profiles of in vitro activated leukocytes were correlated with leukocytes isolated from patients with microbiologically proven infection. In order to do so, different Raman spectra were computed, as discussed above, to differentiate infection vs. control, infection with fungi vs. bacteria, and infection with Gram-positive vs. Gram-negative bacteria. White light images, Raman maps and cell images after Kimura staining are shown in Supplementary Figure S7. To assess the magnitude of the differences in comparison to cell-to cell variations, PCA was performed for each leukocyte sub-population for each case. The respective scatterplots are shown in Figure S3B,D,F, S4B,D,F and S5B,D,F. It has to be noted that there were no patients with pure fungal infection (Supplemental Table S4); thus, panels D, E, and F might look different for a purely fungal infection. Those difference spectra are presented next to each other for in-vitro- and in-vivo-activated leukocytes in Figure 5. Neutrophils (Figure 5A,D,G) and monocytes (Figure 5C,F,I) show similar profiles in their difference spectra compared to leukocytes from patients and after in vitro activation. When comparing the difference spectra of neutrophils between in-vitro-activated leukocyte subpopulations and patient samples, it can be seen that common patterns are present (Figure 5A,D,G). Major contributing Raman bands present in the leukocytes from patients are evidenced in the in-vitro-activated leukocytes, such as the CH_2_ symmetric stretching band at 2842–2856 cm^−1^ and the CH_3_ symmetric stretching band 2930–2954 cm^−1^. When comparing neutrophils infected with Gram-positive vs. Gram-negative bacteria, the differences in the amide I band are revealed in both infection models.

More significant variations are observed for lymphocytes between in vivo and in vitro data, resulting from the small number of cells measured (Supplementary Table S4), such that different lymphocytes subsets, which would present differences in the activation pattern, are not sampled in representative ratios. Furthermore, differences due to the pathogen’s different incubation/interaction times in the experimental settings compared with the patients cannot be excluded. For lymphocytes, patient data with *K. pneumoniae* was not available. Hence, patients with *E. coli* infection were included in infection with Gram-negative bacteria (Figure 5E,H, Supplementary Table S4). Furthermore, no lymphocytes from a patient with infection with only Gram-positive bacteria were available.

Despite the low cell number available to analyze in vivo infected monocytes, some similarities in in vitro and in vivo stimulated monocytes (Figure 5), particularly in CH_3_ symmetric stretching region around 2842–2859 cm^−1^ and 2936–2948 cm^−1^. However, it has to be noted that for monocytes infected with a Gram-positive bacterium only two cells were available from one patient and thus, in-depth interpretation of these results is not possible.

## 3. Discussion

In the current study, distinct immune responses to different pathogens by the three main leukocyte sub-population could be detected using Raman spectroscopy. The observed changes in leukocyte’s Raman spectra due to infection could be assigned to changes in biochemical composition due to cell activation. The Raman band assignment agrees with previous studies that used other methods, e.g., analysis of the transcriptome [48], lipid composition [49], nuclear processes enabling protein-based defense mechanisms [50], or protein associated molecular patterns, as well as changes in cellular metabolism and epigenetic changes [51,52,53,54] to characterize the host response, demonstrating its potential of to characterize activated leukocyte subpopulations.

The in vitro monocyte infection model showed a good performance in detecting the presence of infection and differentiating the cause of infection. Monocytes are professional phagocytes but are more robust and have a longer lifetime compared to neutrophils. Further, monocytes express more toll-like receptors on their surface [55] that are targeted towards bacteria [43] and were shown to present a differential response against Gram-negative and Gram-positive bacteria [45]. This might explain the excellent performance of monocytes in detecting not only the presence of infection but also the type of pathogen causing the infection. In addition, monocytes were selected from the PBMC fraction after attachment to the surface. It has to be noted that activated monocytes attach more easily which might induce a bias for fully activated cells.

For neutrophils, differentiation of the stimulating pathogen, i.e., bacterial vs. fungal, could be achieved from the Raman spectra of neutrophils with a high balanced accuracy of 82% at the sample level. Neutrophils employ different strategies fighting significant pathogens, such as fungi and smaller bacteria. These involve the production of granule proteins for the formation of NETs, phagocytosis, and lysosome acidification [34,47]. These different strategies result in different activation patterns characterized by a different overall biochemical composition revealed in the cell’s Raman spectra. In the present study, a low multiplicity of infection (MOI) of 0.1 was used to keep the influence of phagocytosis on the Raman data low. The current Raman model faces challenges in discriminating neutrophils infected with Gram-positive and Gram-negative bacteria that might result from the heterogeneity between donors as visualized in the PCA plot (Supplementary Figure S2A) and the small numbers of cells investigated. However, another recent Raman study, wherein almost 20,000 neutrophils were analyzed after one hour of pathogen contact, provided reasonably good differentiation [26].

The present study indicated that Raman spectroscopy can detect lymphocyte activation by pathogens. Raman-based differentiation of the cause of infection was only possible with low balanced accuracy in the current data set. Lymphocytes are part of the adaptive immune response and can be divided into different subtypes. Each subtype is called upon for a specific response, based on the pathogen encountered. [36] It is expected that a higher number of analyzed cells—maybe also differentiated according to the lymphocyte subtype—could improve the diagnostic accuracy. However, an in-depth analysis of lymphocyte subtype activation is out of the scope of the current manuscript and will be considered in future work with higher cell numbers.

To investigate the translational potential of the Raman spectroscopic fingerprints, Raman spectra of leukocytes from peripheral blood of patients with infection (presenting in vivo activated leukocytes) have been compared to the ones from in vitro activated leukocytes. Raman spectra of leukocytes from patients were collected on the same Raman device with the same excitation wavelength but without purification of the leukocyte subtype to enable a timely analysis after blood collection. Thus, the Raman spectra of leukocytes from patients were recorded without prior knowledge of the subtypes, leading to insufficient numbers of measured monocytes and lymphocytes as mostly the primary subtype, i.e., neutrophils, were measured. Hence, for only a few patients are Raman spectral data available for lymphocytes and monocytes. However, for neutrophils—and even for monocytes—a good agreement of the Raman difference spectral profile was observed when differentiating infection vs. no-infection and fungal vs. bacterial infection.

## 4. Materials and Methods

### 4.1. Healthy Donor Blood Collection and Leukocyte Subtype Isolation

The study was approved by the local ethics committee (ethic vote 3558-08/12) and conducted following the Declaration of Helsinki. Eighteen milliliters of whole blood was collected from six healthy volunteers (one male, five females, aged between 30 and 40 years) after informed written consent by vein puncture of one forearm under sterile conditions. Neutrophils and peripheral blood mononuclear cells (PBMCs) were isolated by the density gradient centrifugation method. The whole blood was layered on top of Histopaque solutions (Sigma-Aldrich, St. Louis, Mi, USA), having two different densities 1.119 g/mL and 1.077 g/mL layered one above the other in a falcon tube. The falcon tube layered with blood and Histopaque was centrifuged with rcf of 890 g for 35 min at 20 °C. After centrifugation, PBMCs and granulocytes (majority were neutrophils) were collected separately and washed with PBS (Merck, Darmstadt, Germany). Red blood cells were lysed using ACK lysing solution (Lonza Bioscience, Basel, Switzerland), followed by washing and resuspension in RPMI1640 cell culture medium (Merck, Darmstadt, Germany). Isolated cells were counted using a Neubauer chamber. A cell concentration of 5 × 10^6^ cells/mL for both cell populations (neutrophils and PBMCs) were separately reconstituted in RPMI 1680 containing 10% heat-inactivated human serum (Merck, Darmstadt, Germany).

### 4.2. In Vitro Infection Model

Pathogens used for the stimulation of the immune cells were clinical isolates of the Jena University Hospital. *Candida albicans*, *Klebsiella pneumonia*, and *Staphylococcus aureus* were cultured on LB agar (Carl Roth, Karlsruhe, Germany) for 24 h at 37 °C. Bacterial concentrations were determined by plating successive dilutions and counting the colony-forming units (CFU count). Fungal cells concentration was determined by counting the cells using a hemocytometer. For the infection, pathogens were heat-killed at 99 °C for two hours in a dry block heater [56]. Complete heat-inactivation was proven by plating onto LB agar plates. No growth was observed in any case.

PBMCs and neutrophils were cultured in 25 cm^3^ cell culture flasks at 37 °C and 5% CO_2_: (a) in the absence of any pathogens and in the presence of (b) 5 × 10^5^ cells/mL heat-killed *C. albicans*, (c) 5 × 10^5^ cells/mL heat-killed *K. pneumonia*, (d) 5 × 10^5^ cells/mL heat-killed *S. aureus*. Leukocytes were infected with pathogens using an infection ratio (MOI) of 0.1 Neutrophils were stimulated for 3 h and PBMCs for 24 h, and supernatants were used for cytokine measurements. After the incubation period, neutrophils were washed with PBS by centrifugation using 300 g for 5 min at room temperature, followed by chemical fixation for 10 min at room temperature with 4% formaldehyde solution (Carl Roth, Germany) and finally, washed and resuspended in PBS. After incubation of PBMCs, non-adherent lymphocytes were removed from each flask, and adherent monocytes were collected using EDTA/trypsin solution (Sigma-Aldrich, Germany). Both cell populations were washed with PBS and fixed for 10 min at room temperature with 4% formaldehyde solution, followed by washing and resuspension in PBS. A cell concentration of 10^6^ cells/mL, for each cell population and from each treatment, were placed on CaF_2_ slides (Crystal GmbH) pre-coated with 0.2% gelatin (Sigma-Aldrich, Germany) and dried for ~30 min under a sterile hood. Raman spectroscopic measurement was carried out by placing the CaF_2_ slides with dried cells in a Petri dish filled with PBS. Each sample was processed on the same day and the measurement time required for each leukocyte’s subpopulation was approximately 4 h.

### 4.3. Cytokine Measurement

To validate activation of stimulated leukocytes, excreted cytokines were measured in the supernatants. For PBMCs TNF was used [57] and for neutrophils IL-8 [58]. Cytokines were quantified in duplicates by enzyme immunosorbent assays according to the manufacturer’s instructions (Biolegend). The lower detection limits for TNF and IL-8 were 3.9 pg/mL and 15.6 pg/mL, respectively. In addition, the differences observed in the cytokines TNF, and IL-8 concentrations in supernatants of cells stimulated with each pathogen species were compared with unstimulated cells using the Mann–Whitney test.

### 4.4. Patients’ Blood Collection and Leukocyte Isolation

Patients were recruited within HemoSpec trial (DRKS-ID: DRKS00006265) and form a unique subset. The Raman spectra of patients with microbiologically proven infection were used in this study for comparison. Details regarding the number of patients and the underlying cause of infection are summarized in the Supplementary Table S4. Details on study design and blood sample collection have been published [27]. Briefly, blood samples have been collected following standard protocols into Ethylenediaminetetraacetic acid (EDTA) containing tubes (Sarstedt, Nuembrecht, Germany) from patients diagnosed with sepsis (according to sepsis-3 definition), infection without organ failure or inflammation due to non-infectious origin. For Raman spectroscopic analysis, leukocytes were isolated from 500 µL of blood by removing erythrocytes via red blood cell lyses (NH_4_Cl solution at room temperature for ~10 min) followed by centrifugation (300 g, 5 min, RT). The supernatant was discarded, and the cell pellet was resuspended in 4% formaldehyde (Carl Roth, Germany) and incubated at room temperature for 10 min. The fixed cells were centrifuged, followed by washing with PBS, and resuspended in 0.9% NaCl (VWR, Darmstadt, Germany) solution to yield a concentration of 1 × 10^6^ cells/100 µL. The cell suspension was coated onto CaF_2_ slides pre-coated with 0.2% gelatin. The cells were subjected to Raman spectroscopic characterization immediately after drying.

### 4.5. Raman Spectroscopy

The Raman spectra of leukocytes were collected on an upright Raman system (CRM 300, WITec GmbH) equipped with a frequency-doubled Nd-YAD laser (532 nm). A 100 μm optical fiber was guiding the Raman signal to the spectrometer with a 600 g/mm grating and a back-illuminated deep depletion CCD (DV401A-BV-352 cooled to −60 °C, Andor, Ireland). Spectra of leukocytes after in vitro infection were recorded with a 60× water immersion objective (Nikon) with a numerical aperture (NA) 1.0 after excitation with a laser power of 35 mW in the sample plane. Integration time was 1 s per spectrum. From each leukocyte’s subpopulation, ~10 cells (see Table S5) were analyzed using the mapping function with a step size of 0.75 μm. Leukocytes from patients were characterized as dried cells on CaF_2_ slides pre-coated with 0.2% gelatin. The laser power was reduced to 15 mW laser, and a 100x objective with 0.75 NA (Carl Zeiss, Jena, Germany) was used to record the Raman images in scanning mode. Integration time was 1s per spectrum and with a step size of 0.75 μm ~200 Raman spectra were recorded per scan. On average, 20–30 cells were measured per patient.

### 4.6. Identification of Leukocyte’ Subpopulation in Patient’s Samples Using Kimura Staining

The assignment of the leukocyte subpopulation from the patient’s leukocytes was performed after the Raman measurements by Kimura staining. A total of ten microliters of Kimura staining solution (toluidine blue, 0.03% light green SF yellowish, saturated saponin and phosphate buffer, pH 6.7, all from PAA Chemical) were placed on the cells allowed to stand for 5 min. Then, the stained cells were dip-washed gently with distilled water and allowed to dry at room temperature. Finally, the Raman mapped cells were manually relocated, and cell type was assigned based on nuclear morphology by microscopy (Axio Imager Z1, Carl Zeiss micro) (Supplementary Figure S7).

### 4.7. Analysis of Raman Data

Before data analysis, Raman spectra were preprocessed using in-house written scripts in R [59]. Pre-processing involved cosmic spikes removal [60,61], wavenumber axis calibration [62], background correction using the sensitive nonlinear iterative peak (SNIP) clipping algorithm [63], and vector normalization of the Raman spectra. The wavenumber calibration was performed using 4-acetamidophenol spectrum as a standard reference, measured every time before data acquisition. For in-vitro-activated cells, scans of large areas were used, sometimes covering multiple cells per scan with large background areas in-between the cells, so the average of 10 × 10 spectra (=100 spectra) per cell were analyzed in order to avoid noise coming from the background or the edges of the cell. A total of 678 cells from six donors (see Table S5) were analyzed. For in-vivo-activated leukocytes, the average spectrum per cell area (~185 spectra per cell) were used for the analysis. The spectra were considered background if the median intensity of the CH-stretching area was lower than the standard deviation of the silent area in the spectrum. The average spectra of all donors were calculated for each leukocyte sub-population separately. Raman difference spectra were generated by subtracting averaged preprocessed spectra of the respective groups.

Principal component analysis and linear discriminant analysis (PC-LDA) were used to build statistical models. Separate models were built within each leukocyte sub-population to differentiate between (a) infected and non-infected cells, (b) cells infected with fungi and cells infected with bacteria, as wells as (c) between cells infected by Gram-positive and Gram-negative bacteria. To form the respective classes, different infection conditions were combined (e.g., the class “infected” contains all spectra from cells infected with *C. albicans* or *S. aureus* or *K. pneumonia*). Models were cross-validated using leave-one-donor-out cross-validation by leaving out the data from one donor in each iteration of the cross-validation routine to optimize the number of used principal components (PC). Receiving operating curves (ROC) were generated for each donor using the predicted LDA scores obtained within the cross-validation of the PC-LDA model. PC-LDA models were balanced for an unequal number of cells in different groups by equalizing the prior probability values for the analyzed classes in the LDA model. The area under the curve (AUC) was calculated for each donor separately for each leukocyte sub-population. The average AUC over the donors was calculated to evaluate the models at the cell level. The majority vote prediction was obtained for each donor, treatment and leukocyte sub-population, and the balanced accuracy was calculated to evaluate the models at the sample level. The balanced accuracy was calculated as a mean over sensitivity and specificity of the models on the sample level. A schematic display of the Raman data analysis and the decision tree is shown in Supplemental Figure S6.

## 5. Conclusions

In the present study, the ability to identify leukocyte subtypes, independent of their activation state, using Raman spectroscopy has been shown. Further, Raman spectroscopy allows the capturing of pathogen-induced spectral changes within the immune cells, providing an overview of the biochemical changes due to the immune response in defined in vitro infection models. Distinct response of the different immune cell subtypes to pathogens from different kingdoms is reflected in the Raman spectra. Similar spectral profiles of stimulated leukocytes were detected after in vitro stimulation and after in vivo stimulations in patients with microbiologically proven infection. Monocytes provided the most distinct spectral features, neutrophils are highly sensitive and show pathogen-dependent response, and lymphocytes provided well-balanced accuracies despite their heterogeneity in different cell subtypes. The information obtained from Raman spectroscopy in combination with clinical signs holds the potential to be used for the fast and efficient detection of infections and narrow the infection cause. Thus, ultimately, it could help the physicians to select the appropriate treatment regime for the patients.

## Supplementary Materials

The following are available online at https://www.mdpi.com/article/10.3390/ijms221910481/s1.

## Abbreviations

AUC: area under the curve; BA: balanced accuracy; CCD: charged coupled device; DRKS-ID: Deutsche Register Klinischer Studien Identifikationsnummer (German Clinical Trials Register, Identification number); IL-8: interleukin-8; LD: linear discriminant; LDA: linear discriminant analysis; MOI: multiplicity of infection; NA: numerical aperture; NET’s: neutrophil extracellular traps; PBMC’s: peripheral blood mononuclear cells; PBS: phosphate buffered saline; PCA: principal component analysis; rcf: relative centrifugal force; ROC: receiver operating characteristic; RPMI: Roswell Park Memorial Institute Medium; SE: standard error; TNF: tumor necrosis factor.
