# Supplementary material for: Biochemical Analysis of Leukocytes after In Vitro and In Vivo Activation with Bacterial and Fungal Pathogens Using Raman Spectroscopy
Source: Int J Mol Sci. 2021 Sep 28;22(19):10481. doi: 10.3390/ijms221910481 (PMC8508974; doi:10.3390/ijms221910481)
Supplement: Supplementary file 1 [file ijms-22-10481-s001.zip › ijms-1340429-supplementary.pdf]

Biochemical analysis of leukocytes after *in-vitro* and *in-vivo* activation with bacterial and fungal pathogens using Raman spectroscopy

Aikaterini Pistiki[#,1,2], Anuradha Ramoji[#,1,2,3], Oleg Ryabchykov[1,3], Daniel Thomas-Rüddel[2,4], Adrian T. Press[2,4,8], Oliwia Makarewicz[5], Evangelos J. Giamarellos-Bourboulis[6], Michael Bauer[2,4,7], Thomas Bocklitz[1,3], Jürgen Popp[1,2,3,7], Ute Neugebauer[1,2,3,7*]

[#]Equal contribution

[1]Leibniz Institute of Photonic Technology Jena (a member of Leibniz Health Technologies), Albert-Einstein-Str. 9, 07745 Jena, Germany

[2]Center for Sepsis Control and Care, Jena University Hospital, Am Klinikum 1, 07747 Jena, Germany

[3]Institute of Physical Chemistry and Abbe Center of Photonics, Friedrich Schiller University, Helmholtzweg 4, 07743 Jena, Germany

[4]Department of Anesthesiology and Intensive Care, Jena University Hospital, Am Klinikum 1, 07747 Jena, Germany

[5]Institute of Infectious Diseases and Infection Control, Jena University Hospital, Am Klinikum 1, 07747 Jena, Germany

[6]4th department of internal medicine, National and Kapodistrian University of Athens, Medical School, STREET?, Athens, Greece

[7]Jena Biophotonics and Imaging Laboratory, Albert-Einstein-Str. 9, 07745 Jena, Germany

[8] Medical Faculty, Friedrich Schiller University, Bachstr. 18, 07743 Jena, Germany

[#] Corresponding author: ute.neugebauer@med.uni-jena.de, Phone: +49 3641 9390900

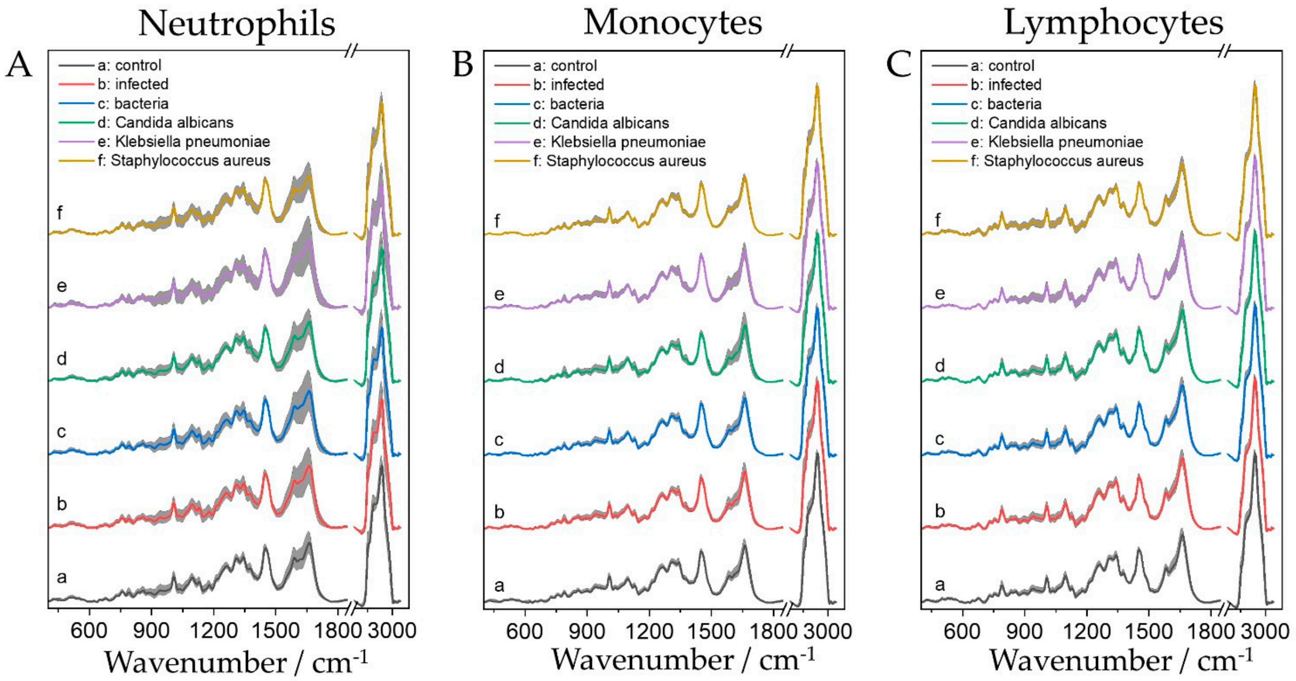

Supplemental Figure S1. Mean Raman spectra of A) neutrophils, (B) monocytes, and (C) lymphocytes. Mean spectra were computed from 6 donors (neutrophils) or 5 donors (monocyts and lymphocytes). The number of used cells can be calculated with the help of Table S5. Raman spectra are shifted along the y-axis for clarity. From bottom to top: a) non-infected, b) infected with pathogens, c) infected with bacteria, d) infected with fungi (*C. albicans*), e) infected with *K. pneumoniae*, f) infected with *S. aureus*.

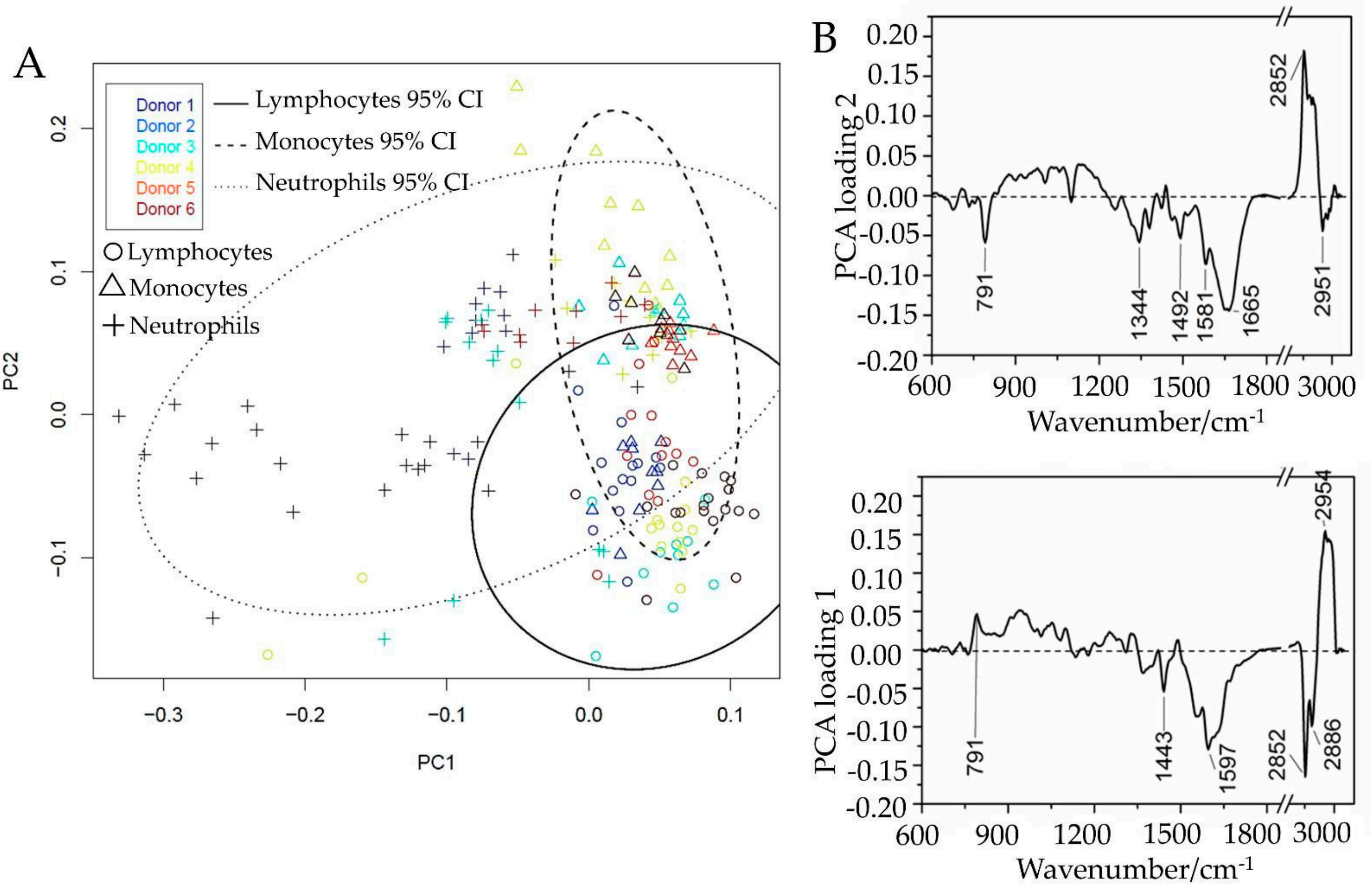

Supplemental Figure S2. Principal component analysis of non-activated leukocytes subpopulations using Raman spectra. (A) PCA score plot showing the distribution of leukocyte subpopulations (lymphocytes, monocytes, and 28 neutrophils) with the ellipses indicating 0.95 confidence interval for each cell population and (B) PC loadings 1 and 2 29 obtained from the PCA analysis show the contributing Raman bands.

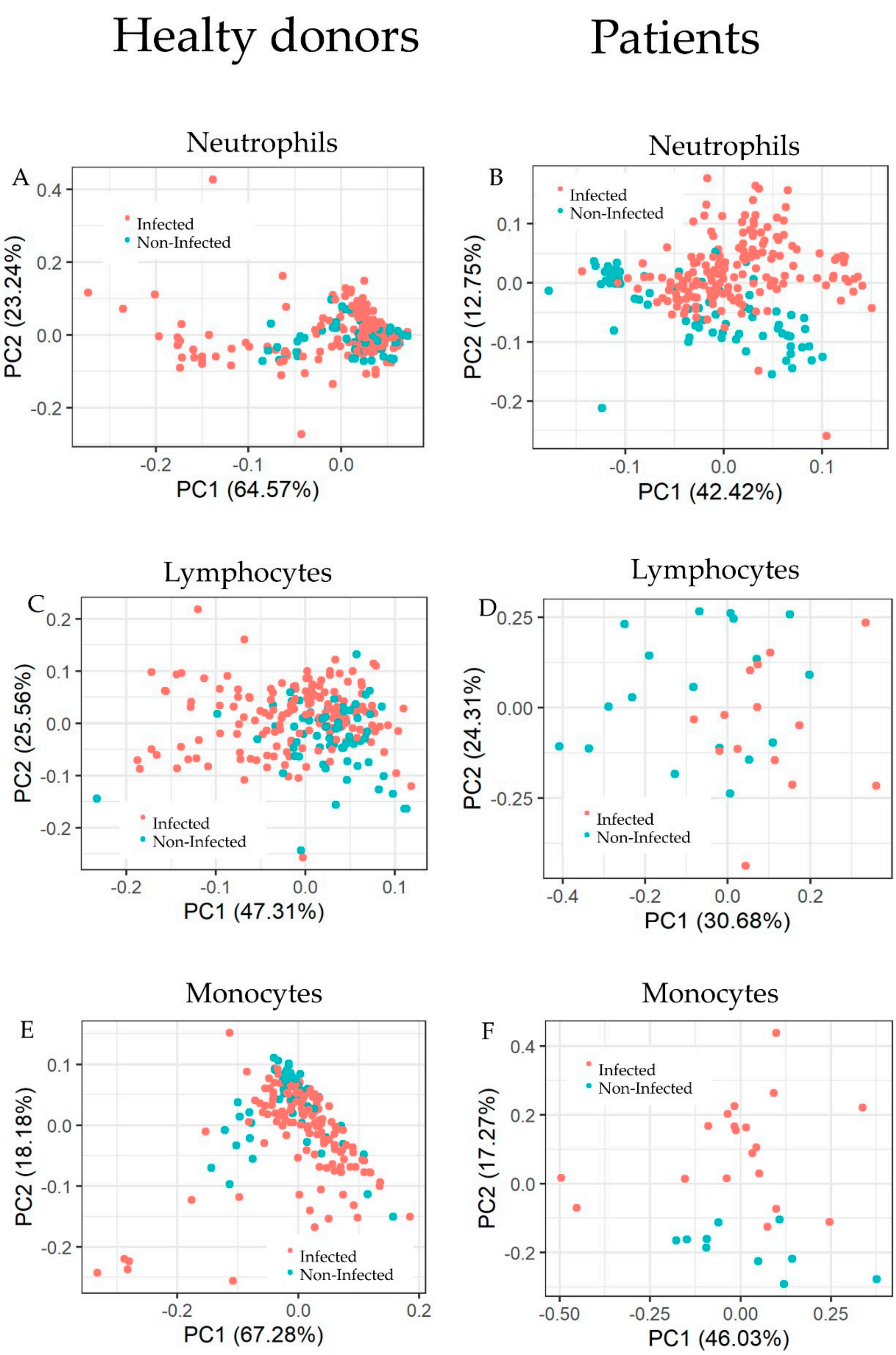

Figure S3. PCA score plots of Raman data to separate infected and non-infected cells. Score plots are shown for different leukocyte subpopulations obtained from healthy donor (left column) and patient (right column) samples. The colors represent infected (pink) and non-infected (cyan) cells. For neutrophils from donor samples, the range of PC scores for infected cells is larger than for the non-infected cells, which points to higher heterogeneity of the infected samples. The differentiation between the infected and non-infected cells is more prominent for patient samples. The separation between infected and non-infected cells appears among patient's lymphocytes visible already in the first PC.

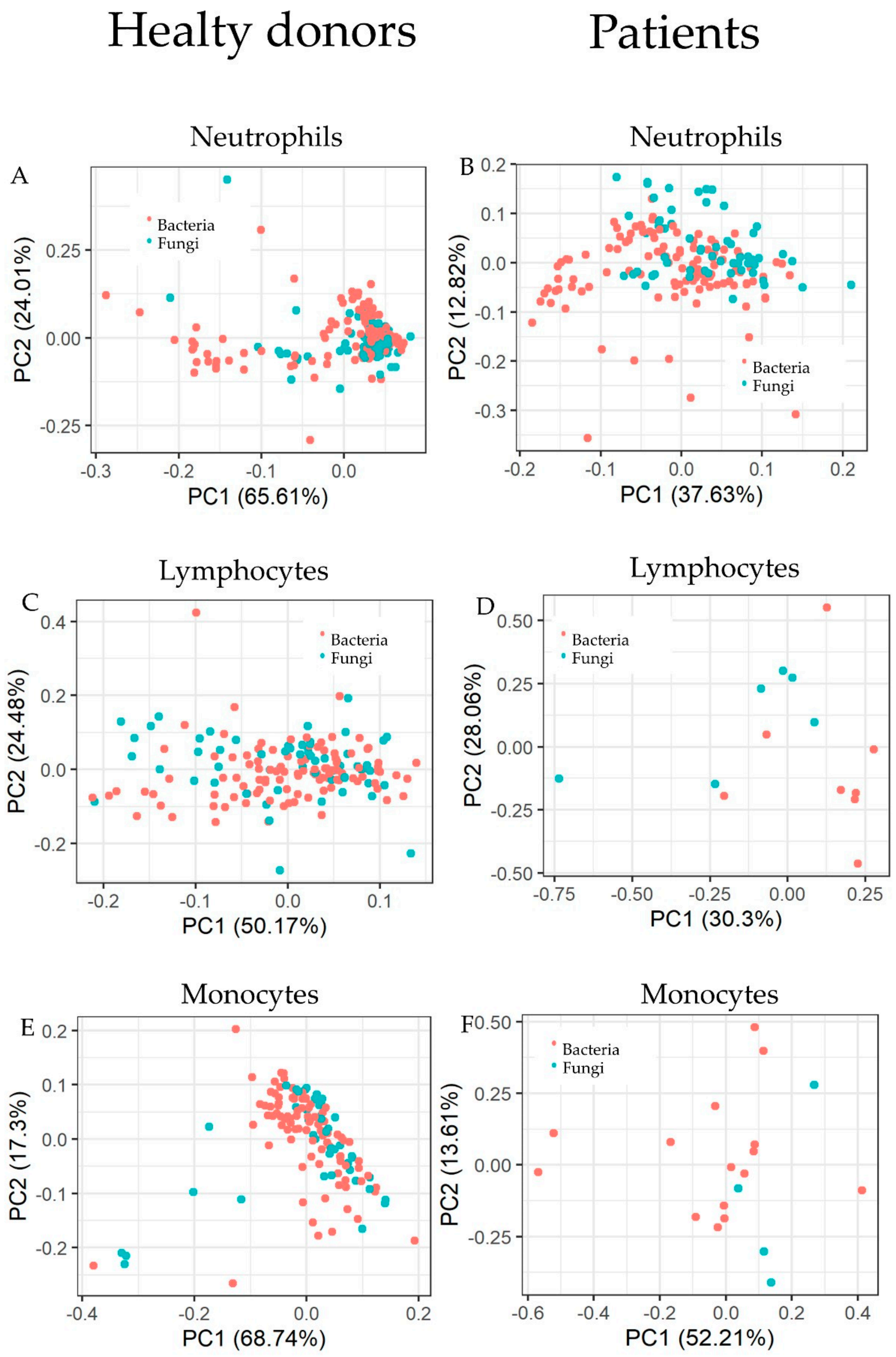

.

Figure S4. PCA score plots of Raman spectra of cells infected with bacteria and fungi. Score plots are shown for different leukocyte subpopulations obtained from healthy donor (left column) and patient (right column) samples. The colors represent cells infected with bacteria (pink) and fungi (cyan). The differences between neutrophils infected with different pathogens is more prominent in patient samples, which is similar to Figure S3. Due to the large difference in number of lymphocytes and monocytes obtained from donor and patient samples, the scatterplots for these two cell subpopulations cannot be reliably compared between each other.

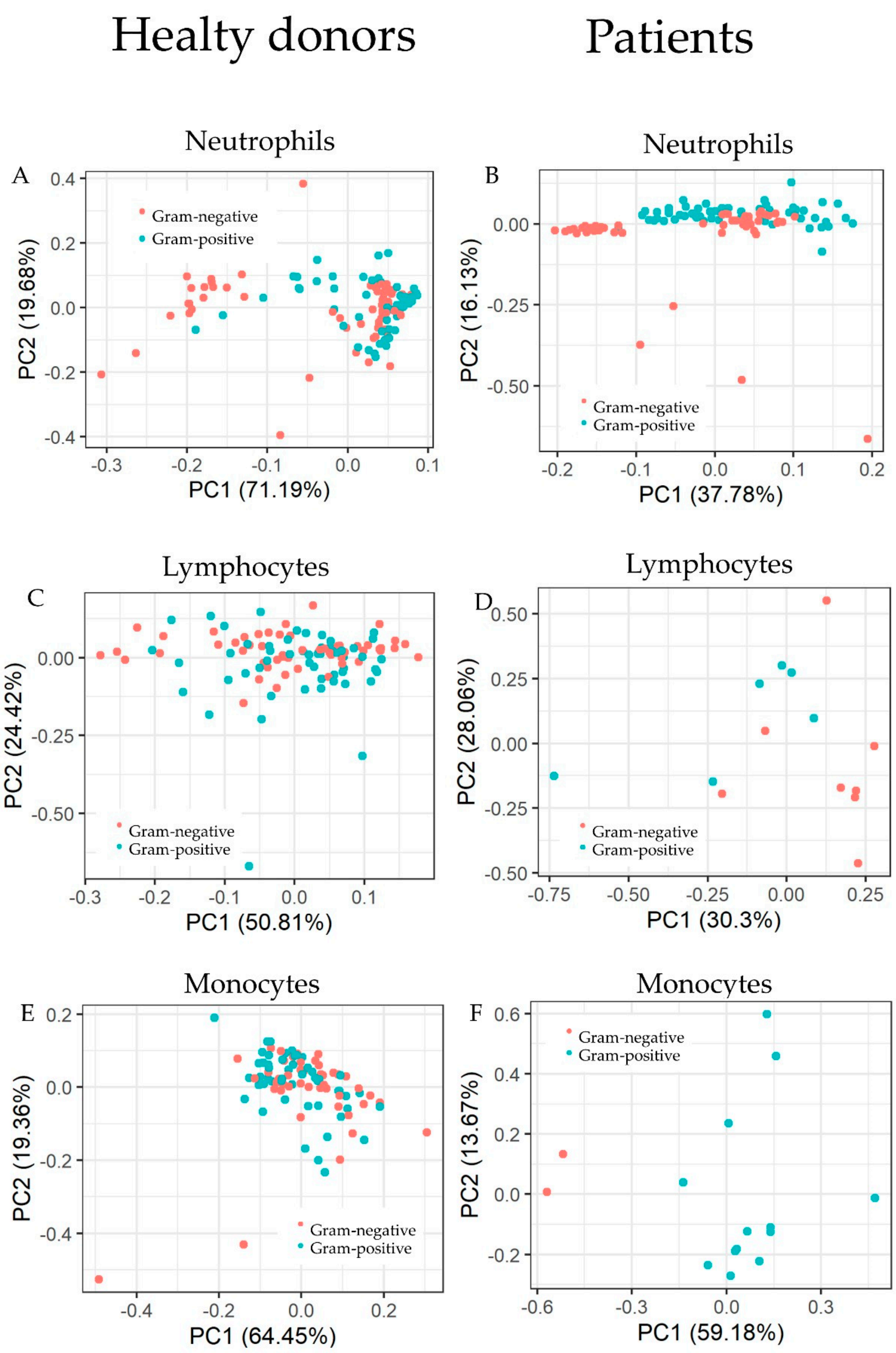

Figure S5. PCA score plots of Raman spectra of cells infected with bacteria. Score plots are shown for different leukocyte subpopulations obtained from donor (left column) and patient (right column) samples. The colors represent cells infected with gram-negative (pink) and gram-positive (cyan) bacteria. Both donor and patient samples infected with gram-negative bacteria show high heterogeneity for neutrophils along the first PC. Good separation of monocytes from the patient infected with gram-negative bacteria should be interpreted carefully, since only two monocytes from a single patient were analyzed in the gram-negative patient samples.

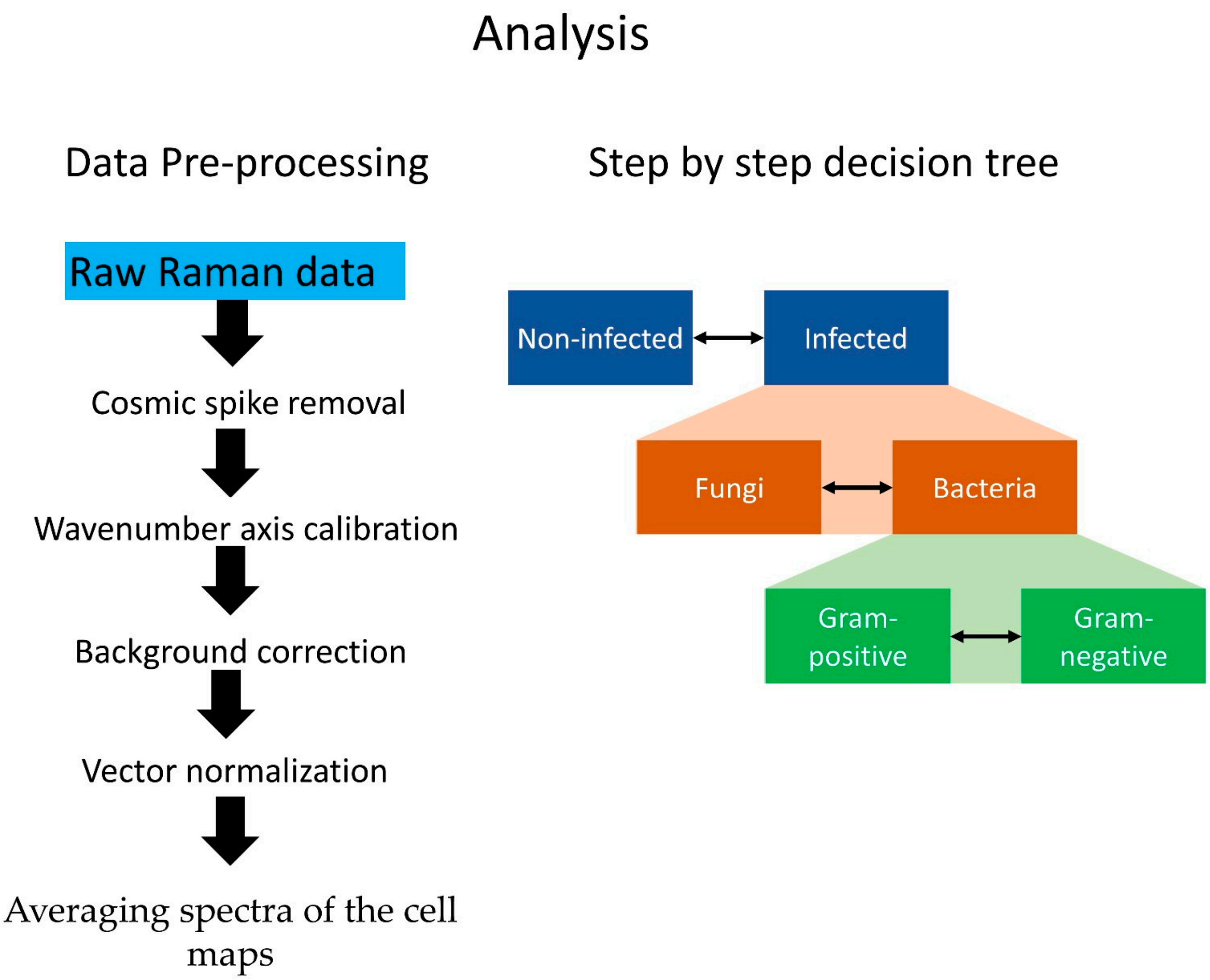

Figure S6. Schematics of the statistical analysis. **Left:** Data pre-processing work-flow. **Right:** Step-by-step decistion tree to differentiate leukocytes (in different treatment groups or from different patients).

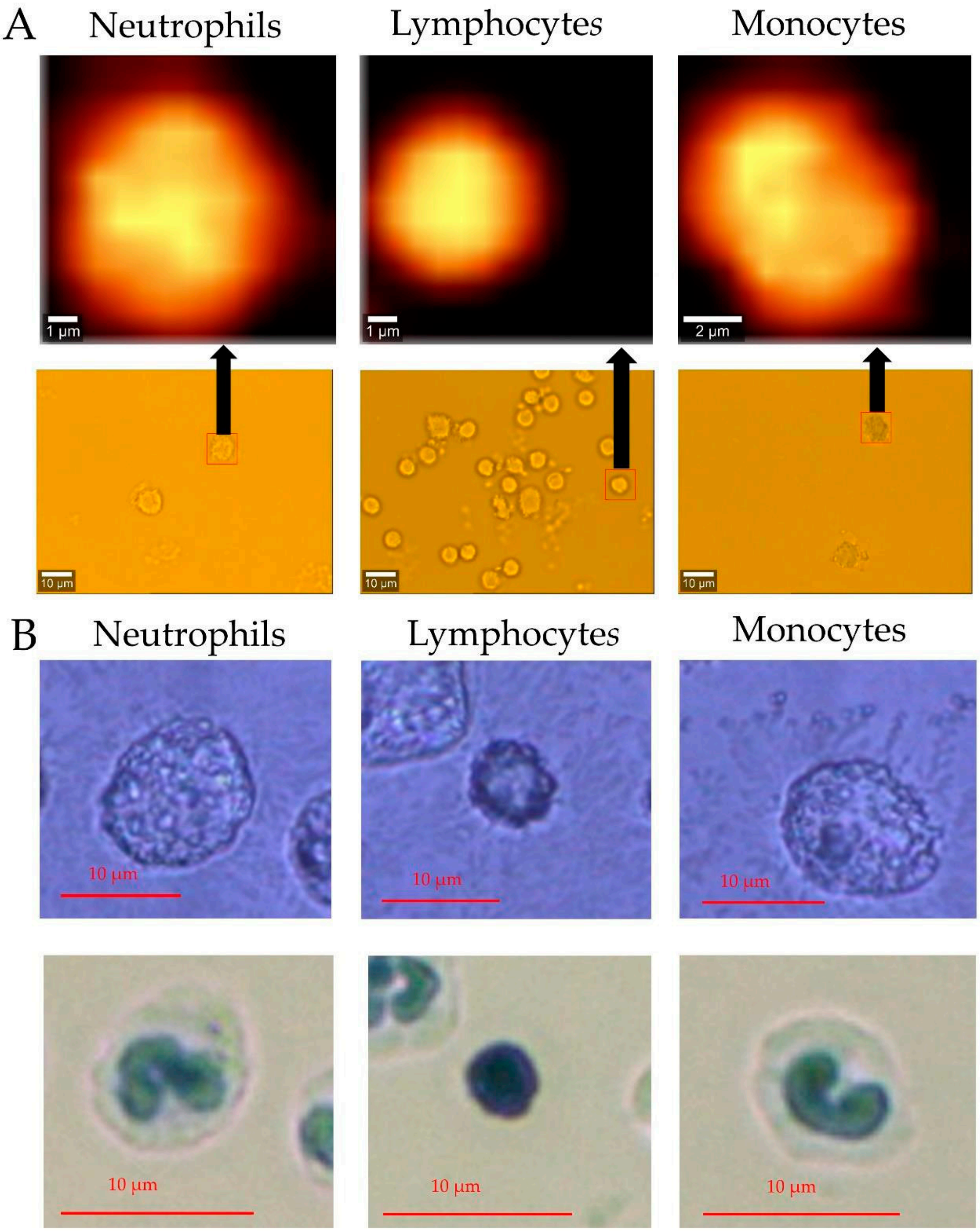

Figure S7. A In-vitro model: Overview white light images are depicted on the bottom. From those images, area scans where selected. Exemplarily, a false color Raman image representing overall Raman intensity of the CH-streching band (2800 cm^{-1}- 3000 cm^{-1}) is shown for each subtype.

B Leukocytes from patients: In the top row white light images of leukocytes isolated from patients after spin-coating onto CaF2 slides are shown. The cells were measured in dried condition to avoid detachment from the slide. After Raman measurements, leukocytes were stained using Kimura. The cells were relocated in the microscope and based on the nucleus shape the leukocytes sub-population were identified. Bottom row shows images of Kimura stained neutrophils, lymphocytes and monocytes from patients which are depicted in the top row.

Table S1. Interpretation of biochemical information from Raman spectra in non-infected and infected (*S. aureus, K. pneumoniae,* and *C. albicans*) lymphocytes and monocytes.

Neutrophils

In-vitro		*In-vivo*				
Non-infected	***Infected***	***Non-infected***	***Infected***	***Raman assignments***	***Biomolecule***	***References***
1348				G(DNA/RNA)	Nucleic acid	(1)
1381						
	1438/43			CH_2CH_3 deformation	All proteins, carbohydrates and lipids	(2)
1589				Cytosine and Guanine of DNA	Nucleic acid	(3)
	1597			Guanine/Adenine	Nucleic acid	(1)
	1633			Amide I (ß-sheet structure)	Proteins	(3)
	2849/52		2846	CH_2 symmetric stretch	Lipids	(4)
2944/47		2948		CH stretching	Proteins	(4)

Lymphocytes

In-vitro		*In-vivo*				
Non-infected	***Infected***	***Non-infected***	***Infected***	***Raman assignments***	***Biomolecule***	***References***
1074				PO_2 backbone C-N str	Nucleic acid	(1, 5)
			754	Thymine, Tryptophan	Nucleic acid	(1, 5)
	791			Phosphate backbone vibrations	Nucleic acid	(1, 5)
	1340/1344			G(DNA/RNA)	Nucleic acid	(1)
	1385					
1443		1436		CH_2CH_3 deformation	All proteins, carbohydrates and lipids	(2)
	1492			C and G of DNA	Nucleic acid	(4)
	1581/1589			Cytosine and Guanine of DNA	Nucleic acid	(1)
		1600		Guanine/Adenine	Nucleic acid	(1)
	1649			Amide I	Proteins	(1, 5)
	1677/1681			NADH and Amide I	Bound and free NADH, proteins	(2, 6)
2856		2854		CH_2 symmetric stretch	Lipids	(4)
2886				CH stretching	Lipids	(4)
2897				CH stretching	Lipids	(4)
			2914	CH stretching	Protein, lipids	(7)
	2947/2951			CH stretching	Proteins	(4)
		2992		=CH asymmetric stretch	Lipids	

Monocytes

In vitro		*In-vivo*				
Non-infected	***Infected***	***Non-infected***	***Infected***	***Raman assignments***	***Biomolecule***	***References***
791				Phosphate backbone vibrations	Nucleic acid	(1, 5)

1005				Phenylalanine	Proteins	(1, 5)
1100				PO_2 backbone C-N str	Nucleic acid	(1, 4)
1235				Amide III (coupled N–H/C–H deformations)	Proteins(7)	(1)
1344/1340				G(DNA/RNA)	Nucleic acid	(1)
	1443			CH_2CH_3 deformation	All proteins, carbohydrates and lipids	(2)
	1556			C-C stretching Tryptophan	Proteins	
1589				C and G of DNA	Nucleic acid	(1)
	1597			Guanine/Adenine	Nucleic acid	(1, 5)
	1629			Amide I (ß-sheet structure)	Proteins	
	2856/2852		2844	CH_2 symmetric stretching of lipids	Lipids	(4)
	2893/2886			CH stretching	Lipids	(4)
2947/2951		2948		CH stretching	Proteins	(4)
2988				=CH asymmetric stretch	Lipids	(4)
		2974		CH_2 symmetric stretch	All proteins, carbohydrates, and lipids	(4)

Table S2. Interpretation of biochemical information from Raman spectra in neutrophils, monocytes, and lymphocytes infected by bacterial pathogens (*S. aureus, K. pneumoniae*) and fungi (*C. albicans)*.

**Neutrophils**						
In-vitro		***In-vivo***				
Bacteria	***Fungi***	***Bacteria***	***Fungi***	***Raman assignments***	***Biomolecule***	***References***
	698					
	764			Trp	proteins	(1, 5)
1010				Phenylalanine	Proteins	(1, 5)
1095				PO_2 backbone C-N str	Nucleic acid	(1, 5)
1265				Amide III random coil	Proteins	(5)
1302				CH_3CH_2 deformation	All protein, lipids, and carbohydrate s	(1)
1335				DNA, Tyr, Trp	Nucleic acid, Proteins	(8)
1451		1438		CH_2CH_3 deformation	All proteins, carbohydrates, and lipids	(2)
	1560			CC stretching Tryptophan	Proteins	(2)
		1578		Guanine/Adenine	Nucleic acid	(1)
	1593			Guanine/Adenine	Nucleic acid	(1)
1625				Amide I (ß-sheet structure)	Proteins	(3)
1669			1662	Amide I (α-helix structure)	Proteins	(1, 5)
2849/2856		2858		CH_2 symmetric stretching	Lipids	(4)
	2934		2930	CH stretching	Proteins	(4)
	2951			CH_3 asymmetric stretching	Proteins	(4)

Lymphocytes						
In-vitro		*In-vivo*				
Bacteria	***Fungi***	***Bacteria***	***Fungi***			***References***
791		788		Phosphate backbone vibrations	Nucleic acid	(1, 5)
			860	Ribose ring vibration	Nucleic acid	(4)
	1010			Phenylalanine	Proteins	(1, 5)
	1104			PO_2- in nucleic acids	Nucleic acid	(1)
		1236		Amide III coupled N–H/C–H deformations	Proteins	(1, 4)
	1256			Amide III coupled N–H/C–H deformations	Proteins	(1)
		1304		CH_3CH_2 deformation	All protein, lipids, and carbohydrates	(1)
	1348			G (DNA/RNA)	Nucleic acid	(1)
		1438		CH_2CH_3 deformation	All proteins, carbohydrates and lipids	(2)
	1459/1451			CH_2CH_3 deformation	All proteins, carbohydrates, and lipids	(5)
1496				C and G of DNA	Nucleic acid	(4)
		1580		Cytosine and Guanine of DNA	Nucleic acid	(1)
	1613			Amino acids	Proteins	(5)
	1621		1622	Amide I (ß-sheet structure)	Proteins	(3)
	1661			Amide I (α-helix structure)	Proteins	(1, 5)
	1677			NADH and Amide I (beta-sheet)	Bound and free NADH	(2, 6)
			1744	C=O ester stretching, non-saturated	Lipids	(9)
		2830		CH_2 symmetric stretching	Lipids	(4)
2852				CH_2 symmetric stretching	Lipids	(4)
2876				CH_3 symmetric stretching	Lipids	(4)
	2883			CH stretching	Lipids	(4)
			2908	CH stretching	lipids	(4)
	2930			CH stretching	Proteins	(4)
**Monocytes**						
In-vitro		*In-vivo*				
Bacteria	***Fungi***	***Bacteria***	***Fungi***			***References***
	675			Guanine (DNA/RNA) C-C twist	Nucleic acid	(4, 5)
	791			Phosphate backbone vibrations	Nucleic acid	(1, 5)

953				Backbone DNA	Nucleic acid	(2)
1014/1010				Phenylalanine	Proteins	(1, 5)
	1100			PO_2 backbone C-N str	Nucleic acid	(1)
1248				Amide III coupled N–H/C–H deformations	Proteins	(1, 4)
1310		1310		CH_3CH_2 deformation	Proteins, lipids, and carbohydrates	(1)
	1381			Guanine, Adenine ring vibrations	Nucleic acid	(10)
1455/1451				CH_2CH_3 deformation	All proteins, carbohydrates, and lipids	(2)
	1492			C and G of DNA	Nucleic acid	(4)
		1548		CC stretching Tryptophan	Proteins	(2)
1593				Guanine/Adenine	Nucleic acid	(1)
1609				Amino acids	Protein	(5)
1661				Amide I (α-helix structure)	Proteins	(1, 5)
1673				NADH and Amide I	Protein	(2, 6)
2859		2842		CH_2 symmetric stretching	Lipids	(4)
2886				CH stretching	Lipids	(4)
	2940		2936	CH stretching	Proteins	(4)
2998				=CH asymmetric stretch	Lipids, fatty acids	

Table S3. Interpretation of biochemical information from Raman spectra in monocytes infected by *K. pneumoniae* and *S. aureus*.

**Neutrophils**						
In-vitro		***In -vivo***				
K. pneumoniae	***S. aureus***	***K. pneumoniae***	***S. aureus***	***Raman assignments***	***Biomolecule***	***References***
		1000		Phenylalanine	Proteins	(1)
		1312		CH_3CH_2 deformation	Proteins, lipids, and carbohydrate s	(1)
	1348			G (DNA/RNA)	Nucleic acid	(1)
		1372		G, A ring vibrations	Nucleic acid	
1443	1438			CH_2CH_3 deformation	All proteins, carbohydrates, and lipids	(2)
		1554		CC stretching Tryptophan	Proteins	(2)
1593				Guanine/Adenine	Nucleic acid	(1)
1637/1641		1648		Amide I	Proteins	(1)
2852		2842		CH_2 symmetric stretching	Lipids	(4)
	2954			CH_3 asymmetric stretching	Proteins	(4)

Lymphocytes

In-vitro		*In-vivo*				
K. pneumoniae	***S. aureus***	***K. pneumoniae***	***S. aureus***	***Raman assignments***	***Biomolecule***	***References***
	791			Phosphate backbone vibrations	Nucleic acid	(1)
			860	Ribose ring vibration	Nucleic acid	(4)
	1100			PO_2- in nucleic acids	Nucleic acid	(1)
		1236		Amide III coupled N–H/C–H deformations	Proteins	(1, 4)
		1304		CH_3CH_2 deformation	All protein, lipids, and carbohydrates	(1)
	1340/1348			G (DNA/RNA)	Nucleic acid	(1)
	1381			Guanine, Adenine ring vibrations	Nucleic acid	
1438/1447		1440		CH_2CH_3 deformation	All proteins, carbohydrates, and lipids	(2)
	1487			C and G of DNA	Nucleic acid	(4)
	1581	1580		Cytosine and Guanine of DNA	Nucleic acid	(1)
	1677/1685			NADH and Amide I (beta-sheet)	Bound and free NADH	(2, 6)
2840/2852				CH_2 symmetric stretching	Lipids	(4)
2883/2886			2886	CH stretching	Lipids	(4)
	2951			CH_3 asymmetric stretching	Proteins	(4)

Monocytes

In-vitro		*In-vivo*				
K. pneumoniae	***S. aureus***	***K. pneumoniae***	***S. aureus***	***Raman assignments***	***Biomolecule***	***References***
791				Phosphate backbone vibrations	Nucleic acid	(1, 5)
	1010	998		Phenylalanine	Proteins	(1, 5)
1104/1100				PO_2 in nucleic acids	Nucleic acid	(1)
1306				CH_3CH_2 deformation	All protein, lipids, and carbohydrates	(1)
1340				Guanine (DNA/RNA)	Nucleic acid	(1)
		1370		Guanine, Adenine ring vibrations	Nucleic acid	
1381						
	1406					
1447				CH_2CH_3 deformation	All proteins, carbohydrates , and lipids	(2)
1492				C and G of DNA	Nucleic acid	(4)

	1556	1550		CC stretching Tryptophan	Proteins	(2)
	1593			Guanine, Adenine	Nucleic acid	(1)
	1625			Amide I (C-O stretching)	Proteins	(5)
	1645			Amide I (alpha-helix)	Protein,	
	1677			NADH and Amide I (beta-sheet)	Bound and free NADH	(2, 6)
2856		2842		CH_2 symmetric stretching	Lipids	(4)
		2872		CH_3 symmetric stretching	Lipids	(4)
2897				CH stretching	Lipids	(4)
	2924			CH stretching	Proteins	(4)
	2940		2946	CH stretching	Proteins	(4)

Table S4. Number of patients, measured (and analyzed) cells per patient and subtypes used to compare *in-vitro* data and *in vivo* data. Leukocyte subtype was assigned by visual inspection of nuclei morphology using Kimura staining after Raman measurement. Thus, not equal numbers of cells were measured in each subpopulation.

	Sterile inflammation *			Infected *						
	number of patients	Pat. ID	Measured (analysed)# cells	Number of patients	Pat. ID	Measured (analysed)# cells	sepsis-3 classifi-cation	Pathogen and focus	Used to differentiate bacteria vs. fungi	Used to differentiate Gram-positive vs. Gram-negative
Neutro-phils	5	1	14 (14)	7	6	21 (17)	sepsis	*S. aureus* / blood and abscess	Bacteria	Gram-positive
					7	11 (10)	sepsis	*E. faecium* + *E.coli* / blood+abdomen	Bacteria	Gram-positive
					8	39 (36)	sepsis	CNS+*Streprococcus spp* / abscess	Bacteria	Gram-positive
		2	22 (19)		9	29 (28)	sepsis	*K. pneumonia* /lung	Bacteria	Gram-negative
		3	9 (9)		10	22 (22)	sepsis	*Lactobacillius spp*+Candida / blood+abdomen	Fungi	-
		4	12 (11)		11	37 (36)	sepsis	*E. faecium* +Candida / abdomen	Fungi	-
		5	44 (23)		12	23 (22)	infection	*K. oxytoca* / urine	Bacteria	Gram-negative
**∑#cells**	**5**		**101 (76)**	**7**		**182 (171)**				
Lympho-cytes	4	1	1 (1)	6	10	4 (4)	sepsis	*Lactobacillius spp* +Candida / blood+abdomen	Fungi	Gram-positive
					11	3 (2)	sepsis	*E. faecium* + Candida / abdomen	Fungi	Gram-positive
					13	2 (2)	sepsis	*Proteus spp.*+ *E. coli* / blood+abdomen	Bacteria	Gram-negative
		2	4 (3)		14	3 (3)	sepsis	*E. coli* / abdomen	Bacteria	Gram-negative
		3	6 (6)		15	2 (2)	sepsis	*E. coli* / urine	Bacteria	Gram-negative
		4	8 (8)		16	1 (1)	infection	*E. coli* / urine	Bacteria	Gram-negative
**∑#cells**	4		**19 (18)**	**6**		**15 (14)**				
Mono-cytes	3	1	3 (3)	6	6	5 (5)	sepsis	*S. aureus* / blood+abscess	Bacteria	Gram-positive
		2	2 (2)		7	4 (4)	sepsis	*E. faecium* + *E.coli* / blood+abdomen	Bacteria	Gram-positive
		4	6 (5)		8	4 (4)	sepsis	CNS+*Streprococcus spp* / abscess	Bacteria	Gram-positive
					12	2 (2)	infection	*K. oxytoca* / urine	Bacteria	Gram-negative
					10	4 (3)	sepsis	*Lactobacillius spp* + Candida / blood+abdomen	Fungi	-
					11	1 (1)	sepsis	*E. faecium* + *Candida* / abdomen	Fungi	-
**∑#cells**	3		**11 (10)**	**6**		**20 (19)**				

* For detailed group assignment see the original publication of the clinical trial (11), sterile inflammation refers to inflammation due to non-infectious origin (e.g. surgery);

# For cases when the whole cell scan was considered background according to the automated quality check described in subsection 4.7 (main manuscript), the scans were excluded from analysis and the actual number of analyzed cells is specified in parentensis.

Table S5. Number of cells measured for each treatment and from each donor. 100 spectra/cell were used for analysis. For donor 6 only neutrophils were analyzed because isolated PBMC's were not enough in number to continue the experiment.

	Leukocytes sub-population	Non-infected	*C. albicans*	*K. penumoniae*	*S. aureus*
Donor 1	Neutrophils	10	10	12	10
	Lymphocytes	14	11	10	12
	Monocytes	10	4	5	10
Donor 2	Neutrophils	13	10	10	10
	Lymphocytes	10	15	11	10
	Monocytes	11	10	10	10
Donor 3	Neutrophils	10	11	10	10
	Lymphocytes	15	10	10	11
	Monocytes	10	10	10	10
Donor 4	Neutrophils	10	11	9	10
	Lymphocytes	13	12	12	10
	Monocytes	10	10	10	10
Donor 5	Neutrophils	11	13	10	13
	Lymphocytes	18	10	11	11
	Monocytes	10	10	10	10
Donor 6	Neutrophils	10	10	19	10

References

1. Neugebauer U, Clement JH, Bocklitz T, Krafft C, Popp J. Identification and differentiation of single cells from peripheral blood by Raman spectroscopic imaging. J Biophotonics. 2010;3(8-9):579-87.
2. Topfer N, Muller MM, Dahms M, Ramoji A, Popp J, Slevogt H, et al. Raman spectroscopy reveals LPS-induced changes of biomolecular composition in monocytic THP-1 cells in a label-free manner. Integr Biol (Camb). 2019.
3. Ramoji A, Ryabchykov O, Galler K, Tannert A, Markwart R, Requardt RP, et al. Raman Spectroscopy Follows Time-Dependent Changes in T Lymphocytes Isolated from Spleen of Endotoxemic Mice. ImmunoHorizons. 2019;3(2):45-60.
4. Pully VV, Lenferink ATM, Otto C. Time-lapse Raman imaging of single live lymphocytes. Journal of Raman Spectroscopy. 2011;42(2):167-73.
5. Notingher I, Verrier S, Haque S, Polak JM, Hench LL. Spectroscopic study of human lung epithelial cells (A549) in culture: living cells versus dead cells. Biopolymers. 2003;72(4):230-40.
6. Chen D, Yue KT, Martin C, Rhee KW, Sloan D, Callender R. Classical Raman spectroscopic studies of NADH and NAD+ bound to liver alcohol dehydrogenase by difference techniques. Biochemistry. 1987;26(15):4776-84.
7. V. PV, M. LAT, C. O. Time - lapse Raman imaging of single live lymphocytes. Journal of Raman Spectroscopy. 2011;42(2):167-73.
8. Walter A, Reinicke M, Bocklitz T, Schumacher W, Rösch P, Kothe E, et al. Raman spectroscopic detection of physiology changes in plasmid-bearing Escherichia coli with and without antibiotic treatment. Analytical and Bioanalytical Chemistry. 2011;400(9):2763-73.
9. Czamara K, Majzner K, Pacia MZ, Kochan K, Kaczor A, Baranska M. Raman spectroscopy of lipids: a review. Journal of Raman Spectroscopy. 2015;46(1):4-20.
10. Neault JF, Tajmir-Riahi HA. Diethylstilbestrol-DNA interaction studied by Fourier transform infrared and Raman spectroscopy. J Biol Chem. 1996;271(14):8140-3.
11. Ramoji A, Thomas-Rüddel D, Ryabchykov O, Bauer M, Arend N, Giamarellos-Bourboulis EJ, et al. Leukocyte Activation Profile Assessed by Raman Spectroscopy Helps Diagnosing Infection and Sepsis. Crit Care Explor. 2021;3(5):e0394.
